# Characterization of LlaKI, a New Metal Ion-Independent Restriction Endonuclease from *Lactococcus lactis* KLDS4

**DOI:** 10.5402/2012/287230

**Published:** 2012-09-30

**Authors:** Abdelkarim Belkebir, Houssine Azeddoug

**Affiliations:** Laboratoire de Biochimie et Biologie Moléculaire, Faculté des Sciences, Université Hassan II-Ain Chock Casablanca, km 8, route d'El Jadida BP 5366, Casablanca, Morocco

## Abstract

Requirement of divalent cations for DNA cleavage is a general feature of type II restriction enzymes with the exception of few members of this group. A new type II restriction endonuclease has been partially purified from *Lactococcus lactis* KLDS4. The enzyme was denoted as LlaKI and showed to recognize and cleave the same site as FokI. The enzyme displayed a denatured molecular weight of 50 kDa and behaved as a dimer in solution as evidenced by the size exclusion chromatography. To investigate the role of divalent cations in DNA cleavage by LlaKI, digestion reactions were carried out at different Mg^2+^, Mn^2+^, and Ca^2+^ concentrations. Unlike most of type II restriction endonucleases, LlaKI did not require divalent metal ions to cleave DNA and is one of the few metal-independent restriction endonucleases found in bacteria. The enzyme showed near-maximal levels of activity in 10 mM Tris-HCl pH 7.9, 50 mM NaCl, 10 mM MgCl_2_, and 1 mM dithiothreitol at 30°C. The presence of DNA modification was also determined and was correlated with the correspondent restriction enzyme.

## 1. Introduction

Restriction-modification enzymes are believed to function as a primitive bacterial “immune” system. They represent the main bacterial protection system against bacteriophage attacks. Restriction-modification systems are composed of two opposing activities: a methyltransferase that protects the host DNA against restriction by methylating the adenine or cytosine residues at certain recognition sites [1, 2] and a restriction endonuclease that recognizes and catalyzes double-strand cleavage of the same sequence if it is unmodified [3, 4]. A large number of restriction-modification systems have been discovered and well characterized during the past few decades; they occur ubiquitously among bacteria and their phages [5]. There are currently 3945 biochemically or genetically characterized restriction enzymes in REBASE and of the 3834 type II restriction enzymes, 299 distinct specificities are known [6]. Based on the cutting position, recognition sequence, cleavage requirements, and subunit structure, R-M systems are mainly classified into four types I, II, III, and IV. The type II R-M systems are the most abundant group of enzymes; they produce double-stranded DNA cleavage within or close the recognition sequence which consists of 4- to 8-defined nucleotides that can be symmetric, asymmetric, unique, or degenerated [7]. 

Most of type II restriction endonucleases show an absolute requirement for divalent metal ions to catalyze in a charge repulsive, polyanionic context the cleavage of the phosphodiester bond, which is one of the most stable bonds in biochemistry [8–10]. Although the physiological metal ion for the bacterial enzymes appears to be the magnesium, they can utilize a variety of divalent cations for in vitro DNA cleavage reaction, including Mn^2+^, Ca^2+^, Fe^2+^, Co^2+^, Ni^2+^, Zn^2+^, or Cd^2+^, depending on the enzyme [3, 4]. X-ray crystallographic analysis of type II restrictions enzymes in different metal-bound states has revealed two DNA cleavage mechanisms in which one or two metal ions are involved [11]. Crystal structure of EcoRI and BglII-like enzymes showed that they contain a single metal-binding site per subunit [12], while the EcoRV-like enzymes showed the existence of two metal-binding sites per subunit [13].

Currently, only three restriction enzymes, BfiI, BmrI, and PabI have been shown to be EDTA-resistant and do not require Mg^2+^ or other divalent metal ions for catalytic activity [14–16]. These enzymes should therefore cleave DNA by a different mechanism. BfiI and BmrI are type IIS restriction enzymes, they recognize the asymmetric sequence, ACTGGG (5/4). BmrI and BfiI belong to the phospholipase D superfamily which includes phosphodiesterases, bacterial nucleases, toxins, and phospholipases because of their conserved catalytic residues HKNE and the metal-independent endonuclease activity. The N-terminal amino acid sequence of BfiI showed similarity to Nuc, an EDTA-resistant nuclease from *Salmonella typhimurium*, which is a member of the phospholipase D superfamily [17]. This paper describes the purification and characterization of a new divalent ion-independent restriction-modification system from *Lactococcus lactis *KLDS4 and its biochemical and biophysical properties.

## 2. Materials and Methods

### 2.1. Enzymes, Bacterial Strains, and DNA Substrates

Commercial restriction enzymes were from New England Biolabs. AmpliTaq Gold DNA polymerase was from Applied BioSystem (California, USA). DEAE cellulose was from Sigma Aldrich; MonoS, MonoQ, and HiTrap Heparin HP 1 mL columns were from Pharmacia. Lambda DNA was purchased from Promega (Madison,WI). DNA plasmids (pUC19, pBR322, and pTrcHisB) were prepared from* E. coli* BL21 (*λ*DE3) using the Sigma GenElute Plasmid Miniprep kit. DNA substrate for divalent metal ions requirement was prepared by PCR reaction of a 500 bp DNA fragment, as described earlier [18]. The concentration of DNAs was estimated by measuring absorption at 260 nm.

Pure strains were isolated from cow milk and grown in MRS (Man Rogosa Sharp, Difco) medium at 30°C without agitation. Identification was made on the basis of gram coloration, morphological analyses, substrates utilization, and degradation of biopolymers using Api 20E (BioMerieux, France). Genomic DNA was isolated and 500 bp fragment of 16S rDNA was sequenced with the following primers 27f AGAGTTTGATCCTGGCTCAG and 519r GWATTACCGCGGCKGCTG. Sequence homologies were searched in the BLASTN database (Basic Local Alignment Search Tool) of the National Center for Biotechnology Information.

### 2.2. Protein Purification

A single colony of *Lactococcus lactis *KLDS4 was used to inoculate 8 L of MRS medium; the culture was grown overnight at 30°C without shaking. The culture was harvested by centrifugation at 7000 g for 10 min at 4°C. The harvested cells were washed with 25 mM Tris-HCl pH 7.5 and frozen at −20°C. Thawed cells were resuspended in (10 mM Tris-HCl pH 7.5, 1 mM EDTA, 10 mM 2-Mercaptoethanol, and 4 mg/mL of lysozyme) and disrupted by 20 cycles of sonication (Bronson Sonifier, USA) of 30 sec each while keeping the temperature around 4°C. The slurry was cleared by centrifugation at 15,000 g for 30 min at 4°C and the resulting supernatant was adjusted to 50 mM NaCl and 1.5% streptomycin sulfate, and then stirred for 30 min at 4°C before centrifugation at 15,000 g for 20 min to remove nucleic acids. Proteins in the supernatant were concentrated with ammonium sulfate to a final concentration of 80%. The protein pellet was dissolved in a minimal volume of buffer B-50 (10 mM Tris-HCl pH 7.5, 1 mM EDTA, 10 mM 2-Mercaptoethanol, and 50 mM NaCl) and dialyzed against the same buffer. The dialyzed protein preparation was applied at a flow rate of 1 mL/min to a 50 mL DEAE-cellulose column that has been previously equilibrated with B-50 buffer. The column was extensively washed at the same flow rate with the same buffer solution. Fractions of 4 mL were collected and those containing the restriction endonuclease activity were pooled. The restriction endonuclease was not retained by the DEAE-cellulose and was found in the flow through. All fractions containing restriction activity were pooled and loaded directly into a Mono Q column. Bound proteins were eluted with a linear gradient of NaCl (100 mM–1000 mM). Fractions showing restriction activity were pooled, diluted, and applied into a Mono S column which was eluted with a linear gradient of NaCl (100 mM–1000 mM). Finally, active fractions eluted from Mono S were combined, diluted, and passed through a HiTrap Heparin HP 1 mL column and eluted with linear gradient of NaCl (100 mM–800 mM). The restriction enzyme was conserved in the storage buffer (10 mM Tris-HCl, 1 mM EDTA, 100 mM NaCl, 50% glycerol) and stored at −20°C. Protein concentration was determined spectrophotometrically at 280 nm.

### 2.3. Size Exclusion Chromatography

To determine the native molecular weight of the restriction endonuclease, size exclusion chromatography was performed with a Sephadex G-200 10/30 column (GE Healthcare, USA), which was preequilibrated with buffer B-100 (10 mM Tris-HCl pH 7.5, 1 mM EDTA, 10 mM 2-Mercaptoethanol, and 100 mM NaCl). LlaKI was loaded onto the column and eluted with the same buffer at a flow rate of 0.5 mL/min. The standards used for setting up the calibration curve were Blue dextran (2000 kDa), Thyroglobulin (669 kDa), B-amylase (200 kDa), BSA (66 kDa), and Carbonic anhydrase (29.4 kDa). Fractions of 0.25 mL were collected and assayed for restriction activity on lambda DNA. 

### 2.4. Denaturing Polyacrylamide Gel Electrophoresis

Sodium dodecyl sulfate polyacrylamide gel electrophoresis (SDS-PAGE) was performed as described previously [19] on one-dimensional 12% polyacrylamide slab gels containing 0.1% SDS. Fractions from each column were loaded on the gel and proteins after electrophoresis were visualized by Coomassie Brilliant Blue staining. The apparent molecular masses of proteins under denaturant conditions were estimated by comparison with the mobility of standard proteins.

### 2.5. Restriction Activity Assays

Unmethylated lambda DNA was used as a substrate for restriction activity assays. The digestion reaction was carried out at 30°C for 1 hour in 30 *μ*L in the presence of 2 *μ*L aliquot of each column fraction. The following buffers were used to find the optimal cleavage condition: buffer 1, 10 mM Tris-HCl pH 7.0, 10 mM MgCl_2_, and 1 mM dithiothreitol; buffer 2, 50 Mm NaCl, 10 mM Tris-HCl pH 7.9, 10 mM MgCl_2_, and 1 mM dithiothreitol; buffer 3, 100 mM NaCl, 50 mM Tris-HCl pH 7.9, 10 mM MgCl_2_, 1 mM dithiothreitol; buffer 4, 50 mM potassium acetate, 20 mM Tris-acetate pH 7.9, 10 mM Magnesium Acetate, and 1 mM dithiothreitol. Reactions were stopped by the addition of 5 *μ*L of the stop buffer (30 mM Tris–HCl pH 7.5, 300 mM EDTA, 0.1% SDS, 25% glycerol, and 0.05% bromophenol blue) and aliquot of 25 *μ*L of each sample was loaded on 1% agarose gel in TAE buffer (40 mM Tris-acetate pH 9.0, 2 mM EDTA) at 700 V/h. 

### 2.6. Substrate Specificity of LlaKI

The recognition sequence of LlaKI restriction endonuclease was deduced from restriction mapping of different DNA substrates of known sequence (lambda DNA, pBR322, pUC19, and pTrcHisB plasmids). Digestion reactions were incubated at 30°C in buffer 2 containing 1 *μ*L of the restriction endonuclease. Double-digestion reactions also were performed in the presence of LlaKI and a second restriction enzyme having a single recognition site in the substrate DNA (PstI, HindIII, NdeI, EcoRV, and BamHI). The DNA fragments produced by digestion were entered into the REB at predictor program available (http://tools.neb.com/REBpredictor/index.php) which predicts possible isoschizomers and the recognition sequence of restriction enzymes [20]. The predicted recognition sequences were compared with the sites generated with single and double restriction endonuclease digestions. 

### 2.7. Metal Ion Requirement of LlaKI Restriction Endonuclease

Divalent metal ion dependence of DNA cleavage by LlaKI was carried out in the presence of different metal ions at various concentrations. Digestion reactions were performed in a buffer of constant ionic strength containing 10 mM Tris-HCl pH 7.9, 0.1 mg/mL bovine serum albumin, 5 mM 2-Mercaptoethanol, 0–50 mM divalent metal ions (MgCl_2_, CaCl_2_, or MnCl_2_), and 50 nM of 500 bp DNA fragment containing one recognition site. NaCl concentration was varied to adjust the ionic strength to 150 mM. DNA cleavage was also carried out in EDTA-buffer (10 mM Tris-HCl pH 7.9, 10 mM EDTA, 0.1 mg/mL bovine serum albumin, and 5 mM 2-Mercaptoethanol). Reactions were started by the addition of 1 unit of metal-free enzyme and incubated for 30 min at 30°C then stopped by the addition of 5 *μ*L of the stop buffer. Aliquot of 25 *μ*L of each sample was loaded and subjected to electrophoresis through 1% agarose gel in TAE buffer (40 mM Tris-acetate pH 9.0, 2 mM EDTA) at 700 V/h. Gels were stained with ethidium bromide (0.5 *μ*g/mL) for 15 min and subsequently destained twice in water for 10 min. Destained gels were photographed using a DOC-PRINT DP-001-FDC gel documentation system (Vilber Loumate, France). One unit of LlaKI restriction enzyme is defined as the amount of enzyme required to completely digest of 1 *μ*g of lambda DNA at 30°C for 1 h in a total volume of 50 *μ*L in buffer 2. 

## 3. Results and Discussion

### 3.1. Purification of LlaKI Restriction Enzyme

**Figure 1:**
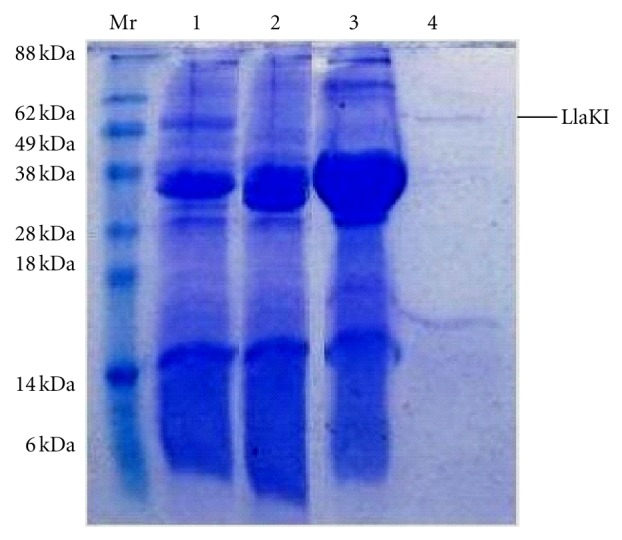
SDS-PAGE analysis of the purification steps of LlaKI. Samples from different steps of the purification procedure were resolved on 12% polyacrylamide gel. Mr: prestained molecular weight marker, line 1: flow-through fraction of the DEAE cellulose column, line 2: Mono Q column fractions pool line 3: Mono S column fractions pool line 4: HiTrap Heparin HP fraction. The line indicates the LlaKI band.

**Figure 2:**
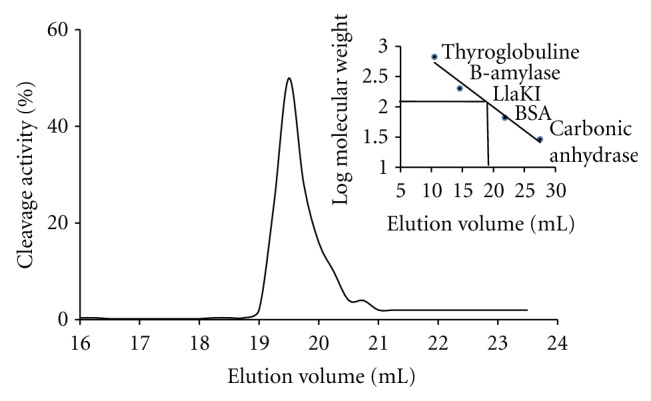
Elution profile from the sephadex G-200 10/30 column. Estimation of native molecular weight of LlaKI was made on the basis of the elution profile of protein standards. Blue dextran (average Mr 2000 kDa) was used to measure the void volume (Vo) of the column and elution volume (Ve) was determined from the UV absorbance at 280 nm for standard proteins and by assay of the cleavage activity for the eluted fractions. Thymoglobulin (669 kDa) Ve: 10.5 mL, B-amylase (200 kDa) Ve: 14.6 mL, Bovine serum albumin (Mr 66 kDa) Ve: 21.85 mL, carbonic anhydrase (Mr 29 kDa) Ve 27.55 mL, and LlaKI (112 kDa) Ve: 19.5 mL.

*Lactococcus lactis *KLDS4 was isolated from cow milk and identified by conventional phenotypic methods and 16S ribosomal RNA (*rRNA*) gene sequencing. Restriction assays on unmethylated lambda DNA showed the presence of a type II restriction endonuclease which was tentatively named LlaKI according to the suggested nomenclature rules [7]. The crude extract of *Lactococcus lactis *KLDS4 was loaded into DEAE-cellulose column and more than 60% of the total protein was retained in the column. The restriction enzyme was found in the flow through and wash volume, which were pooled and applied to a Mono Q column. LlaKI was eluted between 200 and 300 mM NaCl and the obtained protein preparation was nuclease-free. Active fractions were then applied to a Mono S column and the restriction endonuclease was eluted from this column between 400 and 500 mM NaCl. To further purify LlaKI restriction enzyme, active fractions were finally passed through a HiTrap Heparin HP 1 mL column and the enzyme was eluted between 550 and 600 mM NaCl. SDS-PAGE analysis of the columns fractions showed that LlaKI restriction endonuclease has an apparent molecular weight under denaturing conditions of 50 kDa (see Figure 1). To determine the oligomeric state of LlaKI, gel filtration in buffer B-100 was carried out on a Sephadex G-200 10/30 column. The elution profile showed that the native enzyme eluted around 19.5 mL (Figure 2). Based on the calibration curve, we estimated the native molecular weight to be approximately 112 kDa and concluded that LlaKI under the physiological conditions existed as a dimer since the monomeric form is 50 kDa. Many restriction enzymes are dimers of identical subunits, with one active site for each DNA strand. Previous gel filtration studies showed that BfiI restriction enzyme is a dimer in solution in the absence of DNA and when bound to its recognition sequence. However, two dimers could associate transiently during the cleavage reaction to form a tetramer which is too unstable to detect by gel filtration [21]. Like BfiI, BmrI dimerizes to form a functional catalytic site. Structural and biochemical data suggest that homodimer of BfiI cleaves double-stranded DNA in a sequential manner; it first cuts the bottom strand and then undergoes conformational rearrangement to switch its active site to the top strand before cleaving it [22, 23]. PabI, an Mg^2+^-independent restriction enzyme, exists as a dimer like other type II restriction endonucleases that recognize a palindromic sequence. The dimerization of PabI involves interaction between *β*-strands that protrude from the protein core of the monomers, which leads to mutual extension of both *β*-sheets. [16]. The EDTA-resistant DNase Nuc of *Salmonella typhimurium* that lacks sequence specificity also exists as a homodimer in solution; however, its crystal structure shows only one active site, at the subunit interface [24, 25]. 

### 3.2. Recognition Sequence of LlaKI

**Figure 3:**
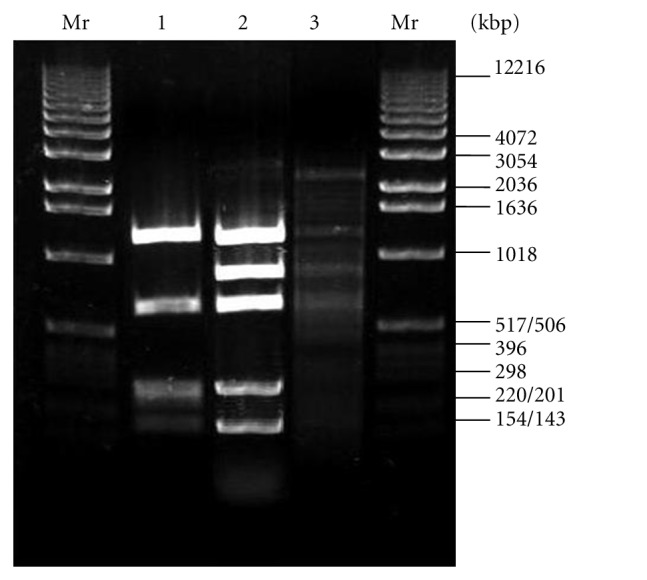
Profile of DNA cleavage by LlaKI on 1% agarose gel. Mr: DNA molecular weight X; line 1: digestion of pUC19 plasmid. Line 2: pBR322 digestion. Line 3: digestion of lambda DNA by LlaKI.

In order to determine the recognition sequence of LlaKI, DNA substrates (lambda, pUC19, pBR322, and pTrcHisB) were digested with LlaKI restriction enzyme at 30°C in buffer 2 containing either 1 *μ*g of lambda DNA or 250 ng of plasmid DNA. Digestion of pUC19 plasmid produced five DNA fragments of approximate sizes 1300, 600, 300, 250, and 190 bp (Figure 3). Restriction analysis using other DNA substrate showed that pBR322 was cut into several fragments of approximately 1200, 800, 600, 300, 150, and a smearing of about 50 bp. The computer-aided search of identical recognition sequences at the mapped LlaKI sites and areas surrounding them using the REBpredictor program [20] revealed only one common sequence GGATG. The predicted LlaKI recognition sequence was in close agreement with the number and sizes of fragments generated by single and double digestion of different DNA in the presence of restriction enzymes with known specificities BamHI, HindIII, NdeI, EcoRV, and PstI (data not shown). Thus, we assumed that LlaKI is another isoschizomer of FokI. 

### 3.3. Restriction Activity Assays

To determine the optimal digestion conditions, we have tested four different buffers in the presence and absence of BSA. LlaKI showed near maximal levels of activity in Buffer 2, 50 mM NaCl, 10 mM Tris-HCl pH 7.9, 10 mM MgCl_2_, and 1 mM dithiothreitol. DNA cleavage by LlaKI restriction enzyme in buffer 1 and 4 resulted in a weak digestion activity. However, digestion in buffer 3 displayed strong nonspecific endonuclease activity and resulted in a smearing pattern on agarose gel. Thus, we assume that LlaKI switched from the specific endonuclease to the nonspecific nuclease upon increasing the salt concentration. BSA did not show any effect on the enzyme activity and cleavage reactions were similar in the presence and absence of BSA. Chromosomal DNA of* Lactococcus lactis *KLDS4 showed methylation protection against its endogenous restriction enzyme, while digestion by restriction endonucleases (BamHI, HindIII, NdeI, and Sau3AI) with different recognition sequences showed a smearing pattern. This data revealed the existence of bacterial methyltransferase activity and confirmed the presence of a complete type II restriction-modification system.

### 3.4. Metal Ion Dependence of DNA Cleavage

**Figure 4:**
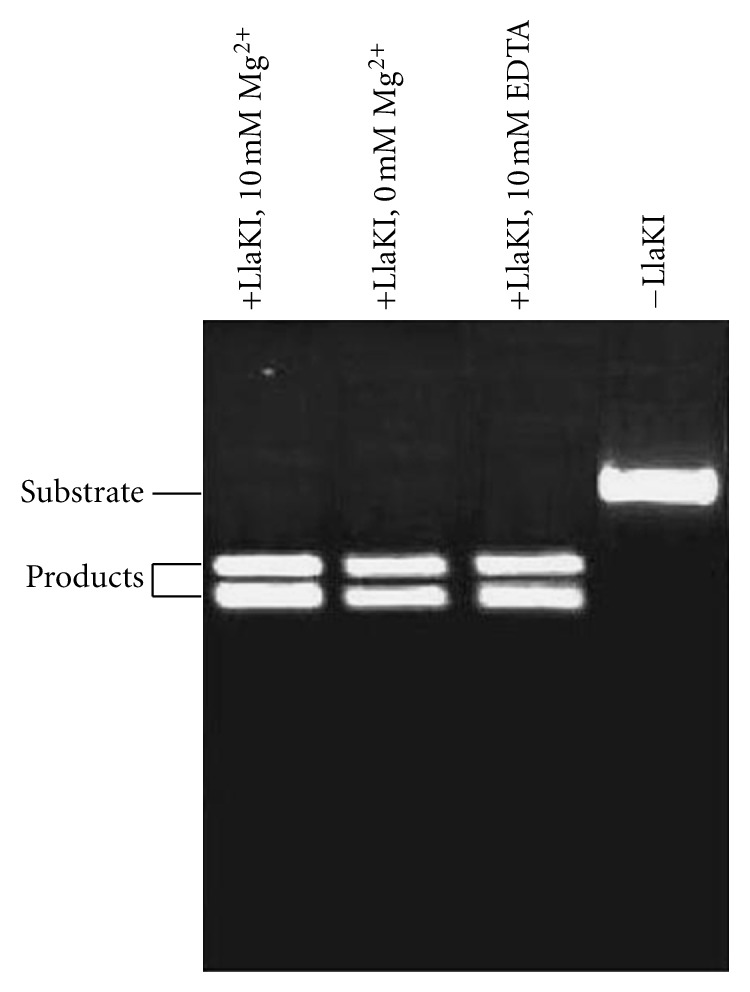
DNA cleavage in the absence of divalent metal ions. Substrate: a 520 bp DNA fragment containing a single recognition site for LlaKI. The restriction enzyme LlaKI cleaved the substrate to yield two DNA fragments of 220 bp and 300 bp. The cleavage reactions were incubated at 30°C for 1 hour.

To determine the metal ion dependence for LlaKI DNA cleavage, we have used a 500 bp DNA fragment that contains one recognition site of the restriction enzyme in the presence of different concentrations of various cofactors (Mg^2+^, Ca^2+^, or Mn^2+^). LlaKI cleaved double-stranded DNA in the absence of divalent metal ions as well as in the presence of Mg^2+^, Mn^2+^, and Ca^2+^. DNA cleavage was also observed even in the presence of 10 mM EDTA. However, concentration above 25 mM Mg^2+^ or 5 mM Ca^2+^ caused inhibition of activity. These results indicate that LlaKI does not require metal ions for specific binding and catalysis of cleavage (Figure 4). Divalent metal ions are required as cofactors for most of restriction endonucleases in particular members of the well-studied PD-(D/E)XK superfamily, to catalyse the cleavage of the phosphodiester bond [9, 10]. The restriction endonucleases MboII from the HNH superfamily [26, 27] and MraI from the GIY-YIG superfamily [28] were also observed to require divalent ions to support DNA cleavage. The natural cofactor for the majority of these enzymes is Mg^2+^, owing to the high negative charge density of DNA and relatively high natural abundance of Mg^2+^ [29, 30]. However, many restriction enzymes are active in the presence of other divalent cations like Ca^2+^, Mn^2+^, or Zn^2+^ [31, 32]. In vitro, the Mg^2+^-dependent enzymes can often mediate the phosphoryl-transfer reactions with Mn^2+^ instead of Mg^2+^. However, such substitution generally leads to reduced efficiency of the enzymatic activity and in many cases Mn^2+^ alters the specificity and the activity of the enzyme. Up to date, only three restriction enzymes BmrI, BfiI, and PabI do not require divalent metal ions to support DNA binding and cleavage [14–16]. Here, we reported a new type II restriction enzyme from *Lactococcus lactis *KLDS4 that cleaves DNA in the absence of divalent metal ions. This result indicated the unique nature of the cleavage reaction by LlaKI restriction enzymes. This result indicated the unique nature of the cleavage reaction by LlaKI restriction enzyme. Thus, it must cleave the phosphodiester bonds by a mechanism different from that of the Mg-dependent restriction endonuclease.
